# On the identification of potential regulatory variants within genome wide association candidate SNP sets

**DOI:** 10.1186/1755-8794-7-34

**Published:** 2014-06-11

**Authors:** Chih-yu Chen, I-Shou Chang, Chao A Hsiung, Wyeth W Wasserman

**Affiliations:** 1 Centre for Molecular Medicine and Therapeutics, Child and Family Research Institute, University of British Columbia, Vancouver, British Columbia, Canada; 2 Graduate Program in Bioinformatics, University of British Columbia, Vancouver, British Columbia, Canada; 3 National Institute of Cancer Research, National Health Research Institutes, Zhunan, Taiwan; 4 Division of Biostatistics and Bioinformatics, Institute of Population Health Sciences, National Health Research Institutes, Zhunan, Taiwan; 5 Department of Medical Genetics, University of British Columbia, Vancouver, British Columbia, Canada

**Keywords:** GWAS, Lung cancer, Regulatory regions, Gene regulation, Transcription factor binding site alteration, Enhancer, Topological domains

## Abstract

**Background:**

Genome wide association studies (GWAS) are a population-scale approach to the identification of segments of the genome in which genetic variations may contribute to disease risk. Current methods focus on the discovery of single nucleotide polymorphisms (SNPs) associated with disease traits. As there are many SNPs within identified risk loci, and the majority of these are situated within non-coding regions, a key challenge is to identify and prioritize variants affecting regulatory sequences that are likely to contribute to the phenotype assessed.

**Methods:**

We focused investigation on SNPs within lung and breast cancer GWAS loci that reached genome-wide significance for potential roles in gene regulation with a specific focus on SNPs likely to disrupt transcription factor binding sites. Within risk loci, the regulatory potential of sub-regions was classified using relevant open chromatin and epigenetic high throughput sequencing data sets from the ENCODE project in available cancer and normal cell lines. Furthermore, transcription factor affinity altering variants were predicted by comparison of position weight matrix scores between disease and reference alleles. Lastly, ChIP-seq data of transcription associated factors and topological domains were included as binding evidence and potential gene target inference.

**Results:**

The sets of SNPs, including both the disease-associated markers and those in high linkage disequilibrium with them, were significantly over-represented in regulatory sequences of cancer and/or normal cells; however, over-representation was generally not restricted to disease-relevant tissue specific regions. The calculated regulatory potential, allelic binding affinity scores and ChIP-seq binding evidence were the three criteria used to prioritize candidates. Fitting all three criteria, we highlighted breast cancer susceptibility SNPs and a borderline lung cancer relevant SNP located in cancer-specific enhancers overlapping multiple distinct transcription associated factor ChIP-seq binding sites.

**Conclusion:**

Incorporating high throughput sequencing epigenetic and transcription factor data sets from both cancer and normal cells into cancer genetic studies reveals potential functional SNPs and informs subsequent characterization efforts.

## Background

Genome wide association studies (GWAS) examine common genetic variants, typically single nucleotide polymorphisms (SNPs), to detect statistical association with a trait across a set of unrelated individuals. Using large-scale SNP genotyping data, these studies compare the genetic makeup between two groups of individuals, those with and without the phenotype or disease of interest. GWAS findings have made important contributions to understanding of numerous disorders.

Once disease associated regions are identified, the challenge is to interpret the potential role of each of the associated SNPs, in order to prioritize candidate functional variants contributing to the phenotype. Around 90% of the variants in GWASdb, a repository of phenotype-associated SNPs identified in GWAS, are situated within intergenic or intronic regions [1]. The analysis and interpretation of such variants are challenging, as sequence-specific functions, such as *cis*-regulatory elements, are present at low density in these regions and have been largely undetermined. The plethora of large-scale data sets generated with human cells creates a new opportunity to probe the relationship between GWAS identified loci and regulatory sequence variants. Both DNase-seq and ChIP-seq datasets generated by consortia such as the ENCODE project [2] have been key. Open chromatin regions defined by DNase I hypersensitivity assays are enriched for the presence of regulatory regions such as promoters and enhancers. Histone modifications such as mono- and tri- methylation of the lysine at position 4 of histone 3 (referred to as H3K4me1 and H3K4me3 respectively) are associated with enhancer and promoter positions [3,4], while acetylation of the lysine at position 27 of histone 3 (H3K27ac) reflects active utilization of the regions [5]. Extensive research into the relationships between diverse chromatin modifications and functional roles of the marked DNA segments is ongoing.

The interpretation of cancer GWAS may be particularly impacted by the study of non-coding regions, as cancer can be considered, at the fundamental level, a disorder of gene regulation. Differential activities of distal regulatory elements, such as enhancers, have been identified at prostate and colon cancer risk variants from GWAS [6-8]. Using H3K4me1 ChIP-seq datasets in colon cancer and normal cells, Akhtar-Zaidi *et al.* identified thousands of variants with loss or gain of H3K4me1, and found them to comprise a signature that is predictive of *in vivo* colon cancer gene expression patterns [6]. Gerasimova *et al.* successfully predicted functional SNPs contributing to asthma, in part by taking into account tissue-specificity of enhancers using epigenetic datasets [9]. Paul *et al.* showed enrichment of SNPs associated with hematological traits within nucleosome depleted regions of hematopoietic cells [10]. One previous study successfully coupled disease associated SNPs to regulatory sequence annotation by pooling and analyzing datasets from multiple cell types to focus on potential regulatory SNPs [11]. As GWAS derived disease-associated SNPs are most commonly found in non-coding regions, incorporating regulatory sequence annotations into the interpretation process is anticipated to further the identification of the causal variations within GWAS loci.

The identification of regulatory sequence variants impacting phenotype has received increasing research attention [12]. Initial methods focused on the identification of mutations that disrupt transcription factor binding sites (TFBS) [13-15]. More specifically, the intersection of GWAS and large-scale regulatory sequence annotation availability has catalyzed the creation of tools focused on the identification or ranking of potential regulatory variants. Ward *et al*. created the online resource tool, HaploReg, to provide annotations for non-coding variants through chromatin states of 9 cell types, conservation and impact on predicted TFBS [16]. The RegulomeDB tool annotates functional variation using a combination of high throughput sequencing (HTS) data, TFBS predictions, eQTLs and enhancer information [17]. Coetzee *et al.* created a functional SNP annotator by incorporating ENCODE TF and histone modification datasets within an R package, FunciSNP, which was subsequently used in a breast cancer GWAS analysis [18,19]. The ChroMoS web server, on the other hand, facilitates SNP prioritization using genetic and epigenetic data, and predicts differential transcription factor and miRNA binding [20].

**Figure 1:**
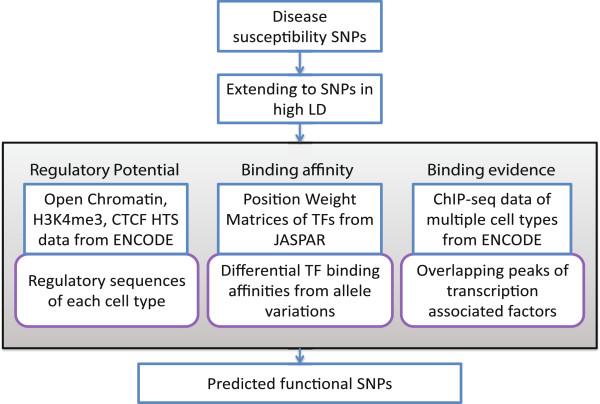
**Overview of regulatory variant discovery workflow.** The analysis workflow takes as input a list of SNPs identified in genome wide association studies, diverse high-throughput sequencing data related to the delineation of cis-regulatory sequences, and position weight matrices (PWMs). The input SNP lists are extended to SNPs in high linkage disequilibrium (LD). Functionality of each SNP is evaluated through the three criteria (regulatory potential, TF binding affinity and binding evidence). The output is a set of candidate variants that display characteristics consistent with a *cis*-regulatory role in the disease process.

In this study, we introduce an approach to the prioritization of regulatory variants within GWAS defined loci. The methods are applied to GWAS cancer susceptibility SNP sets for lung, breast, prostate and colorectal cancers. Based on the observed strong signature of potential regulatory variants and HTS data availability, we focus on the analysis of breast and lung cancer GWAS as models for the prioritization of non-coding functional variants. Our objective is to interpret potential cell type-specific functionality of cancer GWAS SNPs in non-coding regions by incorporating sequence motif information and HTS datasets from the ENCODE project (a workflow overview is presented in Figure 1). We expanded cancer susceptibility SNP sets to SNPs in high linkage disequilibrium (LD). After annotating regulatory sequences based on data sets from cancer and normal cells, we assessed enrichment of the SNPs in regulatory sequences of relevant and non-relevant cell types. We detected significant TF binding affinity differences from position weight matrices (PWM) to narrow the focus toward functional SNPs in regulatory sequences that potentially result in a difference in predicted binding status. ChIP-seq data was also used to identify transcription associated factors (TAFs), including both sequence-specific DNA binding TFs and a broader set of proteins involved in transcription, whose binding may be affected by SNPs. Lastly we examined ENCODE RNA-seq data from tumor and normal cells to report nearby differentially expressed genes. In the breast cancer GWAS and a case study of a published lung cancer meta-analysis [21], we highlight SNPs that fit all criteria and are situated within potential cancer-specific enhancers. Higher order chromatin interaction data was analyzed to infer the potential gene targets of the variants. Overall we prioritized functional SNP candidates by integrating multiple levels of information. The analysis process may serve as a general framework for the investigation of GWAS loci for potential regulatory variants.

## Methods

The bioinformatics analyses were done using R 3.0.2 [22] and Bioconductor 2.13 [23] unless otherwise stated.

### UCSC GWAS SNPs and the corresponding LD80 SNPs

We obtained lung cancer, breast cancer, prostate cancer and colorectal cancer SNP lists from the gwasCatalog table of UCSC database [2] collected by NHGRI [24], and applied the stringent threshold of P < 5×10^−8^ on for genome-wide significance (gwasCatalog downloaded on March 17, 2014; Additional file 1). With the p-value threshold, the Lung.cancer set (in the trait column of gwasCatalog) included SNPs from European GWAS on “Lung adenocarcinoma” and “Lung cancer”. The Breast.cancer set included SNPs from European GWAS on “Breast cancer”, “Breast cancer (male)”, and “Breast Cancer in BRCA1 mutation carriers”. The Colorectal.cancer and Prostate.cancer sets included European GWAS on “Colorectal cancer” and “Prostate cancer”, respectively. We note that some of the studies included meta-analysis. We focused on European descent studies to avoid potential differences in epigenetic status among different ethnicities. All data sets were obtained from published studies, thus no specific ethics approval was required for the study.

To account for potentially biased SNP selection of the SNP arrays, we obtained all SNPs in linkage disequilibrium with the SNPs of interest, using the SNAP webtool [25]. The search distance was limited to 500 kilobases for simplicity, and the r^2^ threshold was set to 0.80 on SNAP for 1000 Genomes project (Pilot 1), Phase III HapMap (release 2), and Phase II HapMap (release 22 and 21) data sets to expand to variants in strongest LD to disease susceptibility SNPs. A union and unique set of variants, which we referred to as LD80, was compiled from these queries for each GWAS SNP set. Since all SNPs in this report were collected from European descent studies, LD80 SNPs were obtained using r^2^ values with the CEU (Utah residents with ancestry from northern and western Europe) population. To eliminate duplicate or obsolete SNPs, the LD80 SNPs were further filtered for unique genomic coordinates in hg19 through BioMart, and analyses were conducted on these LD80 SNP sets.

### High throughput sequencing data from ENCODE

The HTS datasets of H1 embryonic stem (ES) cells, A549 lung cancer cells and its ‘healthy equivalent’ NHLF (Normal Human Lung Fibroblasts), MCF-7 breast cancer and its ‘healthy equivalent’ HMEC (Human Mammary Epithelial Cells), LNCaP prostate cancer and its ‘healthy equivalent’ PrEC (Human Prostate Epithelial Cells), and two colorectal cancer (Caco-2 and HCT-116) cell lines were obtained from the ENCODE project in the hg19 genomic build [2]. We included in our analysis the datasets from H1 ES cells, as GWAS SNPs associated with various cancers have been reported to be enriched in ES cell enhancers [26]. Regions that were in open chromatin as shown by DNase-I hypersensitivity data, and occupied by H3K4me3 and CTCF (an insulator binding protein) were collected along with bound regions for multiple transcription factors. RNA-seq expression data of A549, NHLF, MCF-7, and HMEC cell lines were obtained for expression analysis. Also, the TAF ChIP-seq peaks of the cell lines (A549, H1 ES, HCT-116, MCF-7 cells) were retrieved, and overlapping peaks across the replicates of each TF within each cell line were required for stringency. No TAF data was identified for NHLF, HMEC, LNCaP, PrEC and Caco-2 cells. Detailed information on the retrieved public datasets was provided in Additional file 2.

### Annotation of regulatory sequences

In order to interpret the functionality associated with the SNPs, regulatory sequences were annotated in H1 embryonic stem cells, A549 lung cancer, NHLF lung normal, MCF-7 breast cancer, HMEC breast normal, LNCaP prostate cancer, PrEC prostate normal, Caco-2 and HCT-116 colorectal cancer cell lines. Where data were available, open chromatin regions were specified by DNase I hypersensitive regions from DNase-seq, potential promoters by H3K4me3 ChIP-seq regions, potential insulators by ChIP-seq peaks of CTCF, and finally potential enhancers (pEnh) by DNase I regions lacking H3K4me3 in non-exonic regions.

### Genomic functional categories

The Ensembl transcripts from ENSEMBL GENES 71 were used for annotation information to specify the locations of exons, untranslated regions (UTRs), transcription start and termination sites (TSS and TTS). SNPs within 10 kb upstream of TSSs were labeled as upstream regions, and SNPs within 10 kb downstream of TTSs were labeled as downstream regions. SNPs falling into more than one category were assigned in the priority order from high to low: 5′ UTR, 3′ UTR, exons, genic, upstream, downstream and intergenic.

### Enrichment tests of SNPs in regulatory sequences

In order to test the enrichment of SNPs in regulatory sequences, we randomly drew from the Illumina 660K SNP Array (widely used in GWAS) an equal number of variants with the overall distributions matching three attributes of the LD80 SNP sets: minor allele frequency, GC content in the region +/-500 bps from the variant and distance to the nearest TSS. We separated each attribute into 20 percentile bins and repeated matched drawings 1,000 times. Using these background sets, we determined the distributions of the number of SNPs overlapping each annotated experimental dataset (e.g. open chromatin, H3K4me3, etc.). We then obtained the p-value of each SNP list with respect to each dataset by comparing the true foreground overlapping counts to the background distributions. Multiple hypothesis testing adjustment was conducted using the ‘qvalue’ R package [27].

### Differential TF binding affinity analysis using PWMs

PWM scores have been repeatedly demonstrated to have strong correlation with the sequence specific binding energy of the modeled TF using the PWM scoring procedure described in [28,29]. We computed the TF affinity scores on 30 base pairs up- and down- stream of each SNP with major and minor alleles using PWMs from the JASPAR 2010 database [30]. For binding site prediction stringency, we used 90 out of 130 vertebrate PWMs with information content greater than 10. The best score overlapping each SNP for each TF was retained, and we took the differences between PWM scores of the major and minor alleles to represent the difference in binding affinity of each TF. We randomly selected 10,000 SNPs from Illumina 660K SNP array that had the same single nucleotide variation, similar GC content (+/-500 bps) and similar distance to the nearest TSS as the studied set of SNPs, and used the TF binding affinity differences of these random SNPs as the background (i.e. we matched the proportions of every possible nucleotide substitution, and 20 percentile bins of GC content and distance to the nearest TSS between the LD80 SNP set and the randomly drawn sets). Using such background distribution of score differences for each PWM, we obtained a two-tailed empirical p-value of the difference in TF binding affinity for each LD80 SNP of interest. SNPs with empirical p-values ≤ 0.05 were considered to induce differential affinity for such TF. To further distinguish meaningful differential affinity that potentially infers enhancement or disruption of a TF binding site, we included an additional criterion of PWM scores greater than 80 for either allele.

### Regulatory potential index

For each of the high throughput sequencing data, intersection of regions from replicated data sets was taken, and values were compiled using geometric means across replicates. To obtain a quantitative score for the regulatory sequences, we computed regulatory potential index (RPI) for each cell line by summing the following values for each SNP: the reads per kilobase per million sequencing reads from DNase-seq, the average enrichment values from H3K4me3 and CTCF ChIP-seq data from the ENCODE consortium. The relative regulatory potential was computed as log2 of (RPI_cancer_ + 1)/(RPI_normal_ + 1). In the case of lung cancer, the relative regulatory potential was log2((RPI_A549_ + 1)/(RPI_NHLF_ + 1)). We note that for colorectal cancer, data sets were only available in two cancer cell lines, in such case the relative regulatory potential was computed from the two cancer cell lines and do not reflect comparison between cancer and normal cell lines.

### Case study of a lung cancer meta-analysis

Landi *et al*. published a meta-analysis of lung cancer GWAS [21]. The Lung.Meta SNP list was generated through a meta-analysis of 11 lung cancer GWAS combining histological types with 13,300 primary lung cancer cases and 19,666 controls of European descent. It is a permissive list that is inclusive of all eight SNPs from Lung.cancer collection introduced above. The published set was based on a threshold of P < 8×10^−5^, which we took as reported for a case study to demonstrate how the regulatory analyses can be applied to borderline lung cancer candidate variant prioritization. We note that the published threshold is less stringent than the threshold applied to the UCSC collections, and greater caution is therefore required in assessing the reliability of the candidate.

### Topological domains and chromatin interactions from Hi-C datasets

We downloaded topological associating domains (TADs) and mapped reads of the chromatin interaction datasets in H1 human ES cells and IMR90 fibroblast cells from Hi-C experiments conducted by Dixon *et al*. using the restriction enzyme HindIII [31] (summary files obtained through GEO: GSE35156). We lifted the genomic coordinates of TADs and paired-end reads originally mapped to reference human genome hg18 to hg19 build using the liftOver tool [32]. As the number of paired reads between two genomic regions reflects the degree of interactions, and Hi-C technique is limited by resolution, we counted the numbers of paired reads in 20 kilobase bins and plotted the result using the ‘HiTC’ R package [33].

## Results

### Cancer susceptibility SNPs frequently occur in non-coding regions

**Figure 2:**
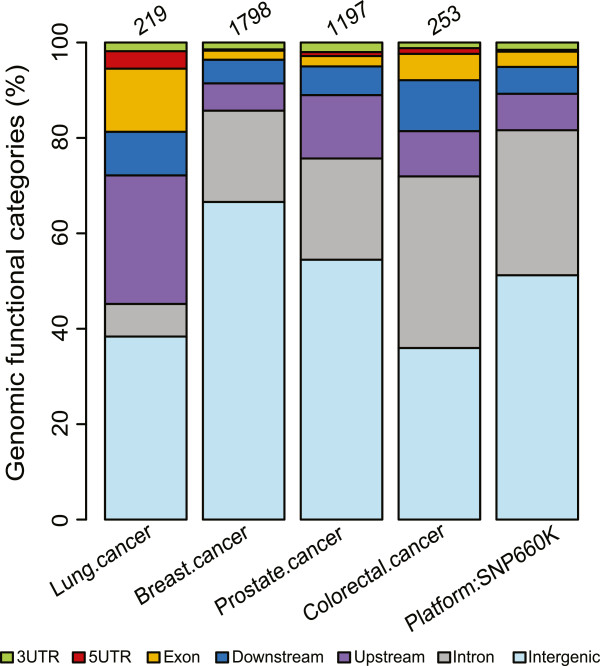
**Distributions of genomic functional categories of cancer GWAS LD80 SNP sets.** For each SNP in the corresponding GWAS LD80 SNP set, the genomic functional category was determined based on genomic annotation, and the overall proportions were shown in the plot. Categories included coding, 5′ untranslated and 3′ untranslated portions of exons, as well as intronic, intergenic and upstream or downstream proximals (within 10 kb of the TSSs or TTSs). The distribution of Illumina 660K SNP array was presented as a background. Numbers above the chart showed the corresponding total SNP counts of each LD80 SNP set.

We retrieved cancer susceptibility SNP sets of multiple lung, breast, colorectal, and prostate cancer studies through the GWAS catalog [24]. Using the SNAP webtool [25], we expanded from the reported SNPs in each set to include those SNPs in high linkage disequilibrium in order to account for potentially biased SNP selection of the SNP arrays. We incorporated such SNPs with an r^2^ greater than 0.8 in the CEU population from either of the 1000 Genomes Project Consortium or the HapMap project (see Methods). We referred to these SNPs in addition to the reported SNPs as LD80, and conducted analyses on the LD80 sets for each GWAS study. In total, we compiled the LD80 sets of 219 lung, 1798 breast, 1197 prostate and 253 colorectal cancer SNPs (Figure 2). To determine how frequently the cancer susceptibility SNPs occur in non-coding regions of the genome, we assessed the distributions of genomic functional categories for each set (Figure 2). We found that over 80% of the SNPs were located in non-coding regions consistently for all LD80 sets.

### Delineating potential regulatory sequences of the genome in different cell types

In order to assess the potential regulatory roles of SNP-containing segments, in both cancer and normal cell conditions, we analyzed a diverse group of features profiled in ENCODE project HTS data sets from cells relevant to the GWAS (detailed in Methods), including DNase-seq, H3K4me3, and CTCF ChIP-seq data. As shown in Additional file 3A, open chromatin regions detected by DNase I sensitivity experiments are largely inclusive of the promoter regions marked by H3K4me3. In agreement with previous literature [34], a large proportion of CTCF bound regions and H3K4me3 marks are shared among cell types, whereas DNase-seq data sets are in greater variation. The CTCF bound regions in NHLF cells are noted to differ from other cell types. We defined regulatory sequences using these markers previously associated with promoters, enhancers and insulators (see Methods). The coverage percentages of the genome for all categories are similar across cell types (Additional file 3B).

### Cancer susceptibility SNPs are enriched in regulatory sequences

**Figure 3:**
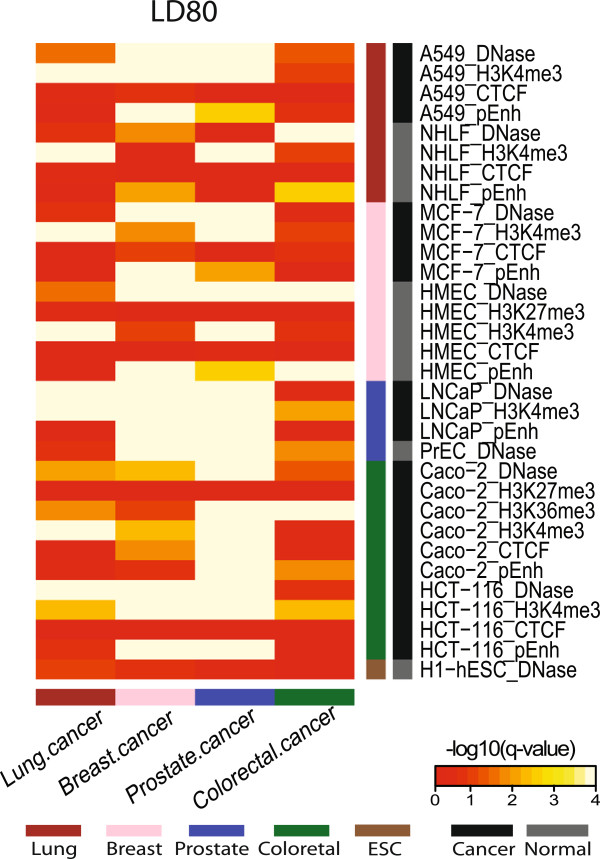
**Heatmap illustration of enrichment of LD80 SNPs in regulatory sequences.** The figure displays the degrees of enrichment significance in regulatory sequences for GWAS SNPs extended to SNPs with r^2^ > =0.80 (LD80). The evaluated LD80 SNP sets are indicated across the horizontal axis. The y-axis indicates the cells of origin and feature data sets that reflects regulatory sequences (all from the ENCODE consortium). Vertical and horizontal side bars are colored according to tissue types and whether it is data from a cancer or normal cell line. Enrichment testing was done by comparing the true foreground overlapping count of each SNP set with each feature data to distributions of overlapping counts by randomly selected SNP sets with matching minor allele frequencies, GC content (+/-500 bps) and distance to the nearest TSSs repeated 1000 times. Multiple hypothesis-adjusted q-values were computed. The enrichment of SNP lists within each feature is colored with a transformed value from multiple hypothesis adjusted q-values: -1x(log10 (q-values +0.0001)). Highly enriched feature and SNP list pairs are colored in yellow, and non-enriched pairs are colored in red.

To investigate the functions associated with regions encompassing the LD80 sets, we tested whether the SNPs are enriched in regulatory sequences both in relevant cells and across tissues for comparison using a total of 32 datasets. Through comparison to randomly drawn SNPs with matching minor allele frequency, GC content and distance to the nearest TSS from the Illumina 660K genotyping array, we found the GWAS LD80 SNPs to be enriched in regulatory sequences of cancer and/or normal cells. Due to the nature of GWAS, the multiple histological types of cancer and the relevancy of the available cell lines to the study, significant enrichment in the cell types and type of regulatory sequences varied between the LD80 sets (Figure 3, Additional file 4). The r^2^ threshold of 0.80 was selected to expand the SNP lists, alternatively a more stringent 0.95 threshold still showed a lesser degree but similar significance in enrichment tests of regulatory sequences (Additional file 5).

Overall, the Prostate.cancer and Breast.cancer LD80 sets showed the most frequent enrichment for regulatory sequences found both within and across tissues, indicating that these SNPs were disproportionately situated within active regions shared among cell types (Figure 3). The Prostate.cancer and Breast.cancer sets were significantly enriched in 22 and 20 categories of regulatory sequences (q-value <0.05), respectively (Additional file 4B,C). The Lung.cancer set was most strongly enriched in the promoter regions of multiple cancer cell lines, MCF-7, LNCaP, Caco-2 and A549 as well as normal cell lines, such as HMEC and NHLF (Figure 3, Additional file 4A). The Breast.cancer set was most strongly, but not exclusively, enriched in the open chromatin regions of MCF-7 breast cancer, A549 lung cancer and HMEC breast normal cells (Figure 3, Additional file 4B). The Prostate.cancer set was enriched most strongly in open chromatin regions of LNCaP prostate cancer, PrEC prostate normal and all other cancer cell lines. Enrichment was also noted for promoter and enhancer regions of multiple cell types as well as CTCF binding sites in Caco-2 cells (Figure 3, Additional file 4C). Due to data availability, regulatory sequences of two colorectal cell lines were examined for colorectal LD80 SNPs. Enrichment was observed most strongly in active transcribed regions marked by H3K36me3 of Caco-2 colorectal cancer cells, open chromatin regions of HMEC and NHLF normal cell lines, and promoter regions of HCT-116 colorectal cancer cells (Figure 3, Additional file 4D). Interestingly, we observed a depletion of the inactive mark, H3K27me3, in Caco-2 colorectal cancer cell line in all four SNP sets.

### Consequence of the SNPs on TF binding affinity scores

Altered binding affinity has been shown to have substantial impacts on the contributions of TFBS [35]. In order to assess the potential impact of SNPs on TF binding, we next scored the predicted differential TF binding affinity between alleles of each SNP using PWMs. We referred to SNPs that result in a significantly higher or lower PWM scores compared to the major alleles as having an increase or decrease of binding affinity for the specific TF, respectively. The impact of a SNP on TF binding affinity was defined by comparing the observed score difference to the distribution of PWM score differences from 10,000 matched randomly selected SNPs from the Illumina 660K SNP array (see Methods). We reported the empirical p-values which reflected the random chance of having such a difference between two alleles at a TF binding site in Additional file 6. We note that a SNP allele can be found with an increase of TF binding affinity for one TF and a decrease for another. The percentages of SNPs with significant (p < 0.05) predicted differential TF binding affinity for at least one TF were 36 for Lung.cancer, 26 for Breast.cancer, 20 for Prostate.cancer, and 30 for Colorectal.cancer. For example, the Sox17 motif was frequently found to have significant differential binding affinity in breast, prostate and colorectal LD80 sets. Our results showed that the SNPs identified in multiple cancer types can alter TF binding affinity as shown by significant changes in PWM scores.

### Prioritizing functional SNPs using regulatory potential and TF binding affinity

**Figure 4:**
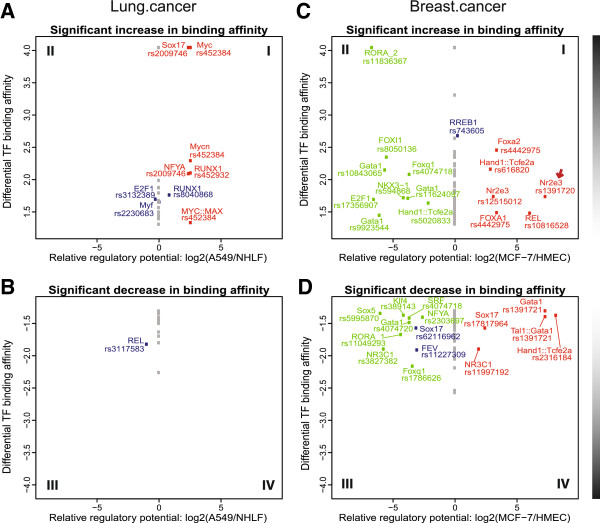
**Differences in regulatory potential and allelic TF binding affinity for Lung.cancer and Breast.cancer LD80 SNPs.** The plots present potentially affected TFBS, with the upper panel **(A & C)** displaying SNPs that confer stronger TFBS patterns in cancer patients with the minor allele while the lower panel **(B & D)** displayed an decrease in TF binding affinity. The x-axis represents the relative regulatory potential, defined as log2 ratio of regulatory potential index between cancer and normal cells plus 1. The relative regulatory potential is indicated as positive for higher regulatory potential in cancer cells (A549 for **A** and **B**; MCF-7 for **C** and **D**) and negative for higher regulatory potential in the corresponding normal cells (NHLF normal lung fibroblasts for **A** and **B**; HMEC breast normal cells for **C** and **D**). The y-axis shows the -1xlog2 transformation of empirical p-values for motif affinity score changes. The data shown on the plot are restricted to PWMs with p-values<0.05 from the two-tailed test, and for visualization purposes, only PWMs with scores > 85 in at least one allele are shown. TFs with an increase or decrease of TF binding affinity where the SNP has non-zero regulatory potential in either cancer or normal cells are labeled along with the corresponding SNP. SNPs with zero regulatory potential index in both cells are represented by gray dots, whereas those with regulatory potential indices >0 in both cells are colored in blue. SNPs with regulatory potential index restricted to a single cell type (cancer or normal cells) are colored in red and green, respectively. In plot **C**, a red arrow indicates a SNP rs1391720 that is discussed in the text. The vertical bar illustrates the degree of difference in TF affinity.

To prioritize functional SNPs, we examined the relative regulatory potential by comparing cancer to normal cell lines where data is available (detailed in Methods) and differences in predicted TF binding affinity by comparing major and minor alleles. We did not restrict the TF affinity analysis to SNPs present in the cancer cell lines we worked with, and we assumed the regulatory potential in the cancer cell lines shows whether regions are active and accessible in the corresponding cancer cells. Figure 4 showed the functional prioritization plot for Lung.cancer and Breast.cancer due to higher data availability, and plots for other LD80 sets were provided in Additional file 7. In Figure 4, the SNP-impacted TFBS in quadrants I were consistent with the presence of a stronger TFBS in regulatory regions preferentially observed in the cancer samples, quadrant II with the presence of a stronger TFBS in regulatory regions preferentially observed in normal cells, quadrant III with the presence of a weaker TFBS in regions preferentially observed in normal cells, and quadrant IV with presence of a weaker TFBS in regions preferentially observed in cancer cells (i.e. loss of a silencing TFBS). The magnitude of relative regulatory potential observed in the Breast.cancer set was higher than that of the Lung.cancer set. We found that an increase of TF binding affinity in the minor allele was not necessarily associated with a gain of regulatory potential in the cancer cell line, and vice versa.

### Prioritizing functional SNPs using all three criteria

**Figure 5:**
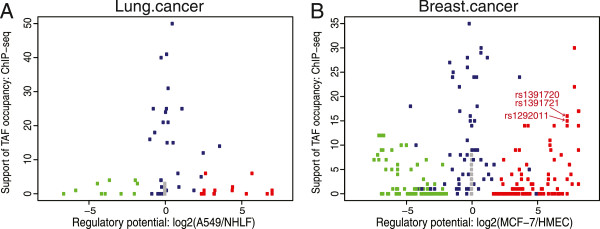
**Visualizing Lung.cancer and Breast.cancer LD80 SNPs with TAF ChIP-seq binding data.** The relative regulatory potential is plotted along the x-axis, as in Figure 4. The y-axis displays the number of TAF ChIP-seq data sets reporting binding in multiple cells examined in this study: A549, H1 embryonic stem, HCT-116, MCF-7 cells. Each dot represents a SNP within the Lung.cancer **(A)** and Breast.cancer **(B)** LD80 lists. SNPs with zero regulatory potential indices in both cells are represented in gray dots, whereas those with regulatory potential in both cancer and normal cells are labeled and colored in blue. SNPs with only regulatory potential observed in cancer or normal cells are colored in red and green, respectively. The red arrows in **B** highlight a set of correlated SNPs, rs1391720, rs1391721 and rs1292011 that overlaps 15 to 16 TAF ChIP-seq peaks. ChIP-seq datasets used are detailed in the supplementary information (Additional file 2).

To further evaluate how TAF occupancy may aid in prioritizing functional SNPs, we inspected the relationship between relative regulatory potential and the number of overlapping TAF occupied regions reported in ChIP-seq data. We gathered available TAF ChIP-seq data from the ENCODE project in the cell types we investigated. Counts of datasets overlapping each SNP were generally higher for SNPs with regulatory potential in both cell types (blue dots), whereas the SNPs with regulatory potential restricted to either cancer or normal cells (red or green dots) were occupied by less TAFs (Lung.cancer and Breast.cancer sets shown in Figure 5, plots for other LD80 sets were provided in Additional file 7). This result is expected as common regulatory regions that are open and accessible in multiple cell types have higher chances of detectable TAF binding.

We listed SNPs with regulatory potential and TF affinity differences as well as TAF binding evidence in Additional file 6. The nearest differentially expressed genes were indicated based on RNA-seq data, where available. Edwards *et al*. compiled a list of functional genetic variants and/or target genes that were identified from GWAS, for which regulatory functions of variants were experimentally verified through diverse methods such as electrophoretic mobility shift assays, reporter assays, and more [36]. As a validation of our prioritization approach, we compared our results to the Edwards’ list (Additional file 8). For the 5 Edwards’ SNPs that were present in the datasets we analyzed, at least one (but never all) of the three criteria was fulfilled (i.e. differential regulatory potential, predicted differential TF binding affinity or overlap with TAF ChIP-seq regions).

For those variants meeting all three criteria, we further applied a threshold of ±5 to the relative regulatory potential score and a threshold of 6 to the number of overlapping TAF ChIP-seq data (both parameter values were set based on the 95^th^ percentile of all examined SNPs). While 12 regulatory variants emerged in the Breast.cancer set, none of the Lung.cancer variants met the thresholds. Within the breast cancer candidates, a set of three highly correlated SNPs stood out among the highest ranking with interesting cancer-related characteristics: rs1292011 (GWAS P = 9×10^−22^[37]), rs1391720 and rs1391721 (highlighted in Figure 5). While all three SNPs had differential TF affinity of different TFs, the rs1391720 showed the strongest differential TF affinity for a nuclear receptor protein, Nr2e3 (an increase from 72.4 to 87.9 in PWM scores; highlighted by a red arrow in Figure 4). These SNPs are located in a gene-sparse region with multiple long intergenic non-coding RNAs (lincRNAs) within 200 kb of distance, and are over 714 kb upstream of the T-box 3 gene involved with developmental processes, *TBX3*. The SNPs are within a potential cancer-specific enhancer observed to be in open chromatin for multiple cancer cell lines such as MCF-7, A549 and LNCaP, but not in normal NHLF, HMEC and PrEC cell lines (Additional file 9). All three SNPs overlap with binding sites of 9 unique TAFs in MCF-7 cells including Sin3Ak-20, GATA3, Rad21, CTCF, HDAC2, HA-E2F1, NR2F2, ZNF217, and the enhancer marking p300 [38].

### A case study: functional interpretation of a borderline lung cancer relevant SNP located in a cancer-specific enhancer

**Figure 6:**
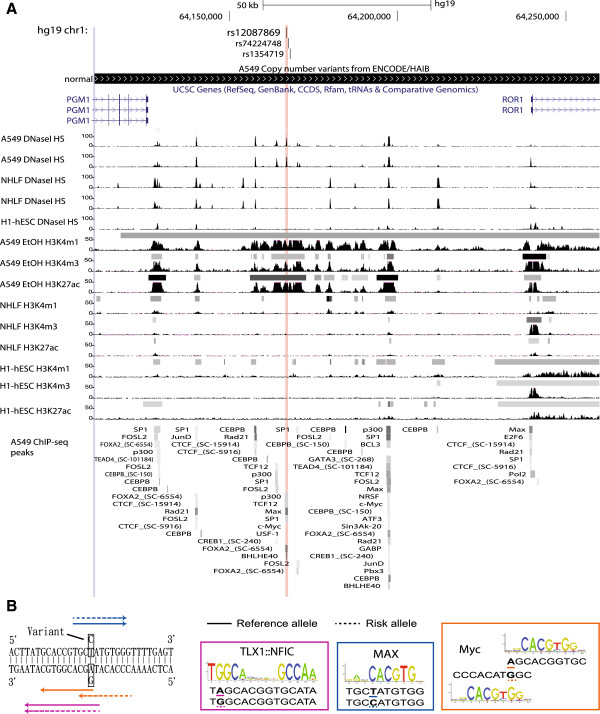
**Annotation features proximal to the rs12087869 SNP location from the Lung.Meta case study.** Part **(A)** depicts annotation related to genetics, epigenetics, and TAF ChIP-seq peaks in proximity to the rs12087869 SNP in A549 lung cancer, NHLF normal and H1 embryonic stem cell lines using the UCSC Genome Browser. The red vertical line highlights the location of the SNP. From the top of the figure, the genetic information includes the locations of the SNP and proximal genes, and copy number status in A549 cells. The chromatin information shows the DNase I hypersensitive sites, occupancy sites of active histone modification marks (H3K4me1, H3K4me3, H3K27ac) in the cell lines. The ChIP-seq section shows the TAF-associated regions in A549 cells where data is available. Peaks of chromatin information and ChIP-seq sections were reported by the ENCODE project with the gray scale color reflecting the magnitude of open chromatin and binding. **(B)** The figure illustrates both strands of the reference sequence within 15 base pairs of rs12087869, and locations of predicted TF binding sites for the reference and risk alleles in solid and dotted lines, respectively. The motif logos for the binding properties of TLX1::NFIC, MAX and Myc are also depicted at rs12087869 risk allele all with increasing binding affinity. The variant within each binding sequence below each logo is underlined, and the predicted Myc binding locations for the reference and risk alleles are different, whereas those of TLX1:NFIC and MAX were the same.

As we did not find regulatory candidates in the Lung.cancer set, we next applied the methodology within a specific case study from a meta-analysis of lung cancer GWAS (Lung.Meta) [21]. We note that the significance threshold (P < 8×10^−5^) applied in the original publication was less stringent, and the reader should apply discretion in evaluating such borderline candidates. Through incorporating the regulatory sequence information as well as differences in TF binding affinity to interpret non-coding SNPs, we identified a specific SNP with interesting cancer-related characteristics (Additional files 10 and 11). The SNP rs12087869 from the Lung.Meta LD80 set was among the highest ranking across all three criteria: regulatory potential differences, predicted TF binding affinity differences, and overlap with TAF ChIP-seq binding sites in A549 cells (Figure 6). It is located 60 kb upstream of a tyrosine-protein kinase transmembrane receptor gene, *ROR1*. The SNP lies within a potential cancer-specific enhancer observed as open chromatin for multiple cancer cell lines such as A549, LNCaP and MCF-7, but not in normal NHLF and HMEC cell lines (Figure 6; Additional file 12). Although open chromatin was detected in the PrEC prostate epithelial cell line, the magnitude was much lower than those of cancer cells. The H3K4me1 and H3K27ac data from the A549 and NHLF cell lines further indicate the region to have enhancer potential in lung cancer cells but not in the normal cells. rs12087869 overlaps with binding sites of eight unique TAFs in A549 cells including c-Myc, Max, and the enhancer marking p300 [38]. In comparison to the non-risk allele rs12087869-T, we found the risk allele rs12087869-C to increase the predicted binding affinity of TLX1::NFIC, MAX and Myc (i.e. the PWM scores rise from 71.6 to 80.2, 69.6 to 82.2, and 78.1 to 90.5, respectively) (Figure 6B; Additional file 13). The A549 cell line does not contain the Lung.Meta associated allele, nor does it display elevated *ROR1* expression relative to NHLF cells (RNA-seq data shown in Additional file 12). Although ChIP-seq data of Myc and Max is not available for the NHLF cells, the presence of overlapping c-Myc and Max A549 ChIP-seq peaks and the predicted increase of binding affinity in the GWAS risk allele are strongly suggestive of a mechanistic role for the rs12087869 SNP elevating risk for lung cancer.

### Inferring potential targets of a SNP using topological domains

**Figure 7:**
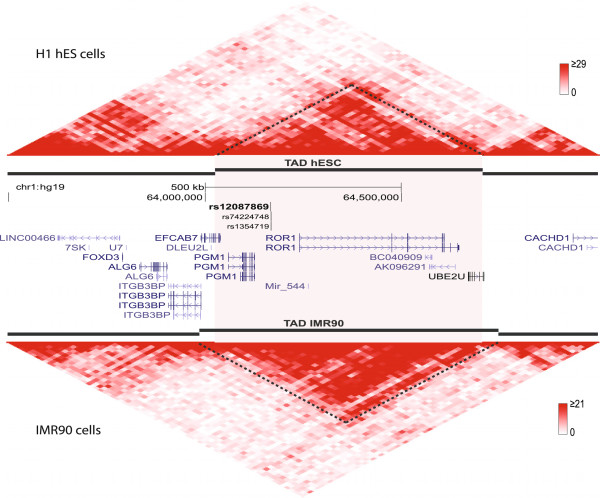
**Two-dimensional heatmap of chromatin interaction in the neighbourhood of the rs12087869 SNP.** The figure shows Hi-C chromatin interaction datasets in H1 human ES cells (upper) and IMR90 fibroblast cells (lower panel) obtained from Dixon *et al.*[31] in the neighbourhood of the rs12087869 SNP. The topological domains (TADs) from both cell types were shown to indicate genomic neighbourhood of stronger within-domain interactions. The heatmap values indicated in a color scale correspond to the number of times that reads in two 20 kb bins were sequenced as a pair, with the red color indicating stronger interaction and white being little or no interaction. The 85 percentile read counts (29 for H1 and 21 for IMR90 cells) were used as the upper limit for the heatmap to avoid color domination of extremely interactive regions. This plot was generated using ‘HiTC’ R package, and the dotted lines were drawn to aid in visualizing the interactive domain in which the SNP is located. The TAD region (from H1 cells) containing the SNP is highlighted in a light pink box.

In order to infer potential gene targets of the enhancer containing the SNP, we used Hi-C chromatin interaction datasets in cells where datasets were available, H1 and IMR90 cells [31]. Enhancers are known that target multiple TSSs, and a recent large-scale enhancer study across human cell types has shown that 40% of inferred TSS-associated enhancers (computed from pairwise correlation of FANTOM5 CAGE data) target at least the nearest TSSs [39]. Such enhancer-TSS interactions vary across cell types, and can be revealed through chromosome conformation capture techniques. Recent studies have shown that the boundaries of highly interactive genomic neighbourhoods (topological associating domains; TADs) were highly consistent across cell types [31,40], whereas interactions between sub-TADs were cell type-specific [41]. Through examining the topological domains and Hi-C chromatin interaction data generated by Dixon *et al*., genes that can potentially be affected by an increase in TF binding affinity of the rs12087869 risk allele include *PGM1*, *ROR1*, *Mir-544*, *BC040909*, *AK096291* and *UBE2U* (Figure 7). Potential targets of the breast cancer susceptibility SNPs that we highlighted in the previous section (rs1292011, rs1391720 and rs1391721) include multiple lincRNAs, *Metazoa_SRP* and *TBX3* (Additional file 14).

## Discussion

Variants in regulatory sequences that affect the transcription rate of target genes can have substantial impact on phenotype. While the interpretation of protein altering differences has progressed rapidly, the identification of *cis*-regulatory modifying mutations remains a challenge. The substantial challenges in moving from GWAS-mapped loci to specific causal variants may be in part derived from this limited capacity to identify regulatory SNPs. We have shown that functional annotation of the regions around non-coding SNPs can contribute to the interpretation of cancer risk alleles arising from GWAS. We observe that cancer susceptibility SNPs are enriched in regulatory sequences in the genome, and can be situated in TF binding sites. Integrating genome-scale data sets and TF binding site analysis into the interpretation process can highlight key SNPs consistent with regulatory roles. Such analysis was used here to highlight SNPs in ChIP-seq supported binding sites within cancer-specific enhancers. This work provides a general bioinformatics approach for the identification of regulatory variants within GWAS identified risk loci.

Previously reported experimentally validated functional SNPs that were evaluated in our study met one or two of our three criteria: regulatory potential, TF binding affinity and TAF ChIP-seq binding. We highlighted a group of three highly correlated SNPs, rs1391720, rs1391721 and rs1292011 in the Breast.cancer set that fit all three criteria. These SNPs are located in a cancer-specific enhancer overlapping 9 MCF-7 TAF ChIP-seq data, and are within the TAD containing multiple lincRNAs and *TBX3* which is situated 714 kb away. Despite the vast distance, a previous study has suggested rs1292011 to be potentially mediating ER-positive breast cancer through an effect on *TBX3* due to significant elevation of expression in plasma from individuals with breast cancer [42]. All three SNPs have differential predicted TF binding affinity of distinct TFs, but rs1391720 shows the strongest significance for an Nr2e3 binding site, which has been previously reported to be an upstream regulator of ESR1 in breast cancer [43].

As a case study, we also investigated the rs12087869 SNP identified as a borderline candidate in a meta-analysis of lung cancer GWAS (Lung.Meta) that fit all three criteria. The SNP is located in a cancer-specific enhancer situated ~60 kb upstream of the cancer-associated *ROR1* gene, which is one of the potential target genes located in the TAD containing the candidate SNP. *ROR1* is highly expressed in early embryonic stages and in diverse cancers such as human breast cancer and B-cell chronic lymphocytic leukemia [44,45]. Another potential target gene, Mir-544, has been reported to be associated with osteosarcoma, gastric cancer, and nasopharyngeal carcinoma [46-48]. The enhancer containing the SNP is marked as active by both H3K4me1 and H3K27ac epigenetics data from A549 lung cancer cells, while these marks of activity are absent in NHLF. The enhancer overlaps ChIP-seq binding peaks of 8 transcription associated factors in A549 cells, including the enhancer marking p300 co-activator and other TFs such as c-Myc, Max, and SP1 which are linked to cancer development and growth [49-51]. Although the cancer cells profiled in large-scale studies (e.g. A549) do not carry the risk allele, the data from the cells indicates that the SNP is situated within a region that is open and accessible in multiple cancer cells (A549, MCF-7, and LNCaP). TLX1::NFIC, MAX and Myc are predicted to have stronger binding affinity in those individuals with the risk allele, highlighting the potential functional role of this SNP in lung cancer.

One of the challenges in the study of regulatory sequences is the relevance of experimental data collected from diverse cells and tissues to the specific cancer cell type. We observe that cancer susceptibility GWAS SNPs are significantly over-represented in regulatory sequences from diverse data sources – the enrichment is not restricted to the specific cancer or normal cell types underlying each GWAS study. Such a finding is not in conflict with a functional role, as a regulatory variant could contribute to the creation of an environment in which a cancer is more likely to form or progress to detectable disease whether it is present in a regulatory sequences of cancer or normal cells. The presence of the TFBS altering change may impact expression in a phenotype-altering manner. From the GWAS SNP sets analyzed, the Breast.cancer and Prostate.cancer LD80 SNP sets showed the highest and most significant enrichment across regulatory regions from multiple cell types. The Lung.cancer and Colorectal.cancer SNP sets had relatively fewer enriched categories of regulatory regions. In particular, we note that the support for the regulatory roles at the breast cancer susceptibility SNP rs1391720 and the borderline lung cancer relevant SNP rs12087869 were provided from cancer-related data collections. Thus, if we had limited the analysis to normal cells, we would not have detected the functional SNP. It is our interpretation that in the search for cancer-related regulatory variants, it is appropriate to consider a range of cell and tissue sources.

This work fits into a spectrum of informatics efforts to better understand regulatory regions in the human genome. Building on high-throughput annotation of genome properties such as epigenetics marks, DNase I hypersensitivity and TF binding, the informatics community has been rapidly creating innovative methods for discrimination of regulatory regions. At the TF affinity level, our initial TF affinity study used only the PWMs from JASPAR 2010, since then, JASPAR 2014 [52] has been made available. Future studies could evaluate alternative sources such as HoCoMoCo [53] which aggregates profiles from multiple resources. At the regulatory sequence level, methods for scoring regulatory potential such as ChromHMM and Segway incorporate diverse data types in order to predict likely regulatory regions [3,54,55].

Recently, three groups have applied informatics methods to predict potential regulatory variants within risk loci from specific GWAS of asthma, breast cancer and blood traits [9,10,18]. Our work is complementary to these studies, showing that GWAS loci from diverse cancer studies are enriched for candidate regulatory SNPs and providing a structured procedure for their identification.

The informatics methods and candidate variants presented here highlight clear opportunities for further work. For example, the cancer susceptibility regulatory SNPs highlighted in the report will require experimental investigation. While *in vitro* binding studies of transcription factors with differential binding affinities could be pursued, it has been well established that computational predictions of binding affinity are highly correlated with experimental measurements [56]. Therefore, we think longer-term it will be most appropriate to obtain relevant cancer cells and normal cells with the specific risk allele and measure TF binding in vivo, expression of potential gene targets (inferred from the Hi-C interaction data and topological domains), and the small-scale chromosome conformation capture in the presence or absence of the candidate regulatory variation. Beyond the specific candidate, a key challenge for the field is the computational prediction of the relationship between enhancers and specific promoters, which would allow for targeted experimental approaches.

## Conclusions

Cancer GWAS SNP sets extended with high linkage disequilibrium are over-represented in *cis*-regulatory sequences active in cancer and normal cells. The degree of over-representation varied across assessed GWAS. Notably, such enrichment of the cancer susceptibility SNPs in normal cells highlights the potential contribution of the variants to the earliest phases of tumorigenesis. Analysis of the differential TF binding affinity between the regular and risk alleles reveals functional SNPs with potential disruption or enhancement of TF binding. We highlight candidate regulatory SNPs, one with borderline lung cancer susceptibility and three with significant breast cancer susceptibility, within cancer-specific enhancers with multiple overlapping TAF binding sites and significant increase in TF binding affinity scores with the risk allele. The methods constitute a framework to interpret functionality of GWAS loci candidate regulatory SNPs based on epigenetics, regulatory potential and TF binding affinity properties.

## Abbreviations

GWAS: Genome wide association study; SNP: Single nucleotide polymorphism; HTS: High throughput sequencing; TF: Transcription factor; TAF: Transcription associated factor; PWM: Position weight matrix; TFBS: Transcription factor binding site; LD: Linkage disequilibrium; TAD: Topological associating domain.

## Competing interests

The authors declare that they have no competing interests.

## Authors’ contributions

All authors contributed to the design of the study. CYC implemented the analysis. ISC and CAH advised about GWAS analysis of cancer risk, and provided feedback on the predictions generated. CYC and WWW wrote the manuscript. WWW supervised the project. All authors read and approved the final manuscript.

## Pre-publication history

The pre-publication history for this paper can be accessed here:

http://www.biomedcentral.com/1755-8794/7/34/prepub

## Supplementary Material

Additional file 1 **Sources of GWAS SNP lists used.** The table lists the literature (PubMed ID) and sources from which we obtained the corresponding GWAS SNP lists. All SNPs passed a genome-wide significance threshold of P < 5×10^−8^, so we used and listed only the studies with at least one SNPs passing the threshold from the gwasCatalog table on UCSC. The Lung.cancer set included SNPs from European GWAS on “Lung adenocarcinoma” and “Lung cancer” (in the trait column of gwasCatalog). The Breast.cancer set included SNPs from European GWAS on “Breast cancer”, “Breast cancer (male)”, and “Breast Cancer in BRCA1 mutation carriers”. The Colorectal.cancer set included European GWAS on “Colorectal cancer”. The Prostate.cancer set included European GWAS on “Prostate cancer”. Click here for file

Additional file 2 **Additional HTS data from ENCODE used to indicate regulatory functions.** The table lists the feature information, name, data type, cell type and data source of each high throughput sequencing dataset we obtained from the ENCODE project. Click here for file

Additional file 3 **An overview of the data depicting the percentages of overlapping regions between regulatory sequences among cell lines.** The heatmap in part A shows the pair-wise percentages of overlapping regions for every feature pair. Features from associated cell types in our study are included. The strongest overlap is indicated in red and the weakest in blue, as depicted in the colour key. Features are clustered according to similarity in overlaps, and are labeled with the cell line names followed by the feature names. The “pEnh” term refers to putative enhancers. The percentage of the human genome covered by each feature is shown in part B. Click here for file

Additional file 4 **Enrichment of GWAS SNPs in all HTS data surveyed.** This illustration provides an alternative presentation of the enrichment result shown in Figure 3. Figures exhibit the overlap counts of LD80 SNPs obtained from Lung (A), Breast (B), Prostate (C) and Colorectal (D) cancer GWAS with regulatory sequences in all cell types examined. The x-axis indicates the cells of origin and feature data sets that reflects regulatory sequences (all from the ENCODE consortium). The y-axis shows the number of SNPs in the set that are within each category of regulatory sequences. The “pEnh” term refers to putative enhancers. The red dots represent the actual overlapping counts of the SNP sets with regulatory sequences, and the boxplots represent the distributions of overlapping counts by randomly selected SNP sets with matching minor allele frequencies, GC content (+/-500 bps) and distance to the nearest TSSs repeated 1000 times. Multiple hypothesis-adjusted q-values lower than 0.05 are noted above the boxplots. Click here for file

Additional file 5 **Heatmap illustration of enrichment of LD95 SNPs in regulatory sequences.** The figure displays the degrees of enrichment significance in regulatory sequences for GWAS SNPs extended to SNPs with r^2^ > =0.95 (LD95). The x-axis represents the LD95 SNP sets, and the y-axis represents data features from all cell types examined. Vertical and horizontal side bars are colored according to tissue types and whether it is data from a cancer or normal cell line. The enrichment of SNP lists within each feature is colored with a transformed value from multiple hypothesis adjusted q-values: -1x(log10 (q-values +0.0001)). Highly enriched feature and SNP list pairs are colored in yellow, and non-enriched pairs are colored in red. Click here for file

Additional file 6 **Results for functional prioritization of LD80 SNP sets.** We list the prioritization results of all LD80 SNP sets examined in the study in multiple tabs of the excel file. The columns include the information on the SNPs, regulatory potential in relevant cancer and normal cell types (where data available), motif affinity differences (significant TFs concatenated with commas), overlap with TAF ChIP-seq data, information on the nearest gene, and nearby differentially expressed transcripts according to RNA-seq data (where data available). Log2 ((RPKM_cancer_ + 1) / (RPKM_normal_ + 1)) were computed for TSSs within 200 kb up- and down- stream of each SNP, and SNPs with greater than 2 fold difference in expression between the relevant cancer and normal cells were reported. Click here for file

Additional file 7 **SNP prioritizing plots of Prostate.cancer and Colorectal.cancer LD80 SNPs.** The file includes plots on differences in regulatory potential and allelic TF binding affinity (A-D) as well as TAF ChIP-seq data (E-F) for Prostate.cancer and Colorectal.cancer LD80 SNPs in addition to the Lung.cancer and Breast.cancer LD80 SNP sets plotted in Figures 4 and 5. Click here for file

Additional file 8 **Comparison to previously reviewed functional genetic variants and their target genes.** The table summarizes the comparison of our prediction results to the potential causal variants listed in the review by Edwards *et al.* 2013. A proportion of SNPs were not included in the input of our analysis. All SNPs that were included in our study met one or two (not all three) criteria in regulatory potential, TF binding affinity or TAF ChIP-seq binding. Click here for file

Additional file 9 **Annotation features proximal to the rs1391720 SNP location from Breast.cancer LD80 set.** The figure depicts annotation related to genetics, epigenetics, and TAF ChIP-seq peaks in proximity to the rs1391720 SNP in MCF-7 breast cancer and HMEC breast normal cell lines using the UCSC Genome Browser. The red vertical bar highlights the location of the 3 SNPs. From the top of the figure, the genetic information includes the locations of the SNPs and copy number status in MCF-7 cells, the SNPs are located in a gene desert. The chromatin information shows the DNase I hypersensitive sites in multiple cell types, occupancy sites of promoter marks in MCF-7 cells and active histone modification marks (H3K4me1, H3K4me3, H3K27ac) in HMEC cells. The ChIP-seq section shows the TAF-associated regions in cells we examined (where data is available). Hotspot of chromatin information and peaks in ChIP-seq section were reported by the ENCODE project with the gray scale color reflecting the magnitude of open chromatin and binding. Click here for file

Additional file 10 **SNP prioritizing plots of Lung.Meta LD80 SNPs from the case study.** The file includes plots displaying differences in regulatory potential and allelic TF binding affinity (A&B) as well as TAF ChIP-seq data (C) for Lung.Meta LD80 SNPs corresponding to Figures 4 and 5. Click here for file

Additional file 11 **Results for functional prioritization of Lung.Meta LD80 SNP set.** We list the prioritization results of the Lung.Meta LD80 SNP set. The columns include the information on the SNPs, regulatory potential in lung cancer and normal cell types, motif affinity differences (significant TFs concatenated with commas), overlap with TAF ChIP-seq data, information on the nearest gene, and nearby differentially expressed transcripts according to RNA-seq data (where data available). Log2 ((RPKM_cancer_ + 1) / (RPKM_normal_ + 1)) were computed for TSSs within 200 kb up- and down- stream of each SNP, and SNPs with greater than 2 fold difference in expression between the lung cancer and normal cells were reported. Click here for file

Additional file 12 **Open chromatin features in other cell lines and expression data around rs12087869.** The figure shows the open chromatin (DNase-seq) features in MCF7, HMEC, LNCaP and PrEC cell lines around the rs12087869 SNP. The RNA-seq signals on the plus DNA strand in A549 and NHLF cells are displayed. Both *ROR1* and *PGM1* genes are expressed in both cell types, and are not differentially expressed comparing between cancer and normal cells (as reported in Additional file 6). Click here for file

Additional file 13 **An exhaustive list of differences in TF binding affinity for Lung.Meta LD80 SNPs.** The table is a flat file of all PWMs predicted on each Lung.Meta LD80 SNP with the JASPAR 2010 PWM IDs, PWM scores in reference and risk alleles, and empirical p-values of the difference. Click here for file

Additional file 14 **Two-dimensional heatmap of chromatin interaction in the neighbourhood of the rs1391720 SNP.** The figure shows Hi-C chromatin interaction datasets in H1 human ES cells (upper) and IMR90 fibroblast cells (lower panel) obtained from Dixon *et al*. [31] in the neighbourhood of the rs1391720 SNP and two other SNPs in high LD. The SNPs overlapping the potential cancer-specific enhancer and over 16 TAF ChIP-seq data are labeled in the middle panel along with genes from UCSC and transcripts from Ensembl that include long non-coding RNAs. The topological domains (TADs) from both cell types were shown to indicate genomic neighbourhood of stronger within-domain interactions. The heatmap values indicated in a color scale correspond to the number of times that reads in two 20 kb bins were sequenced as a pair, with the red color indicating stronger interaction and white being little or no interaction. The 85 percentile read counts (22 for H1 and 18 for IMR90 cells) were used as the upper limit for the heatmap to avoid color domination of extremely interactive regions. This plot was generated using ‘HiTC’ R package, and the dotted lines were drawn to aid in visualizing the interactive domain in which the SNP is located. The TAD region (from H1 cells) containing the SNP is highlighted in a light pink box. Click here for file
