# Supplementary figures and images for: On the identification of potential regulatory variants within genome wide association candidate SNP sets
Source: BMC Med Genomics. 2014 Jun 11;7:34. doi: 10.1186/1755-8794-7-34 (PMC4066296; doi:10.1186/1755-8794-7-34)

A

Heatmap: Overlapping regions in percentage

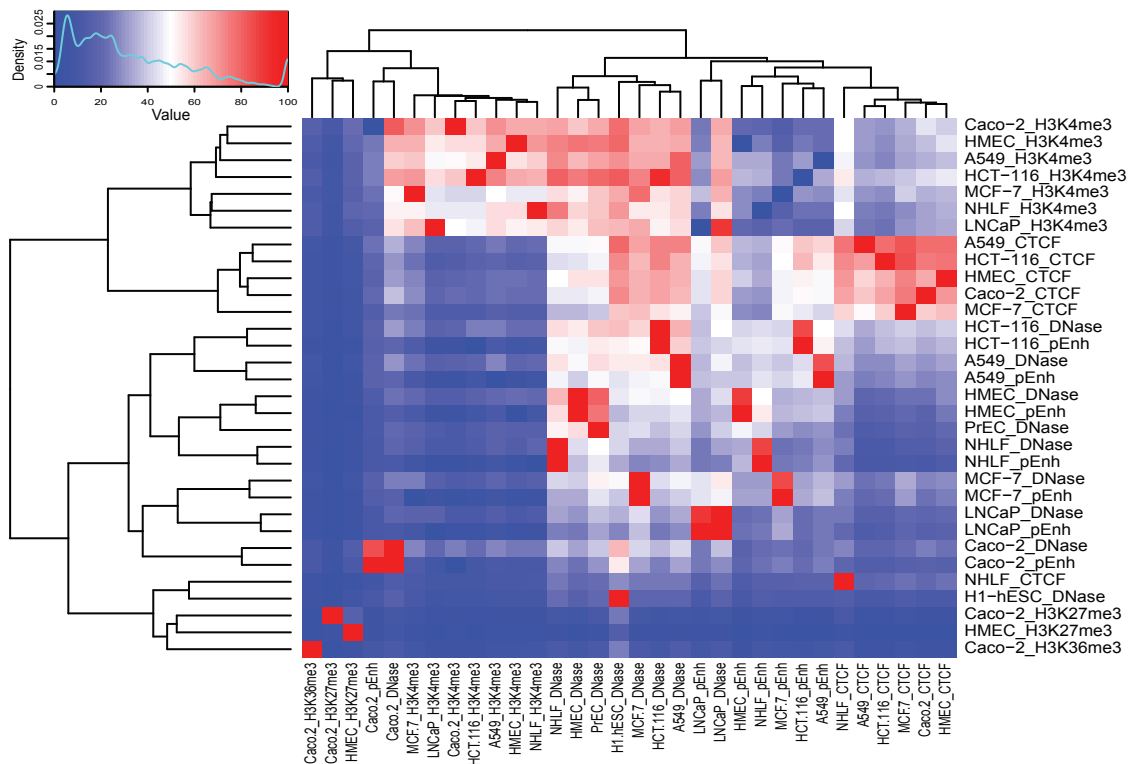

B

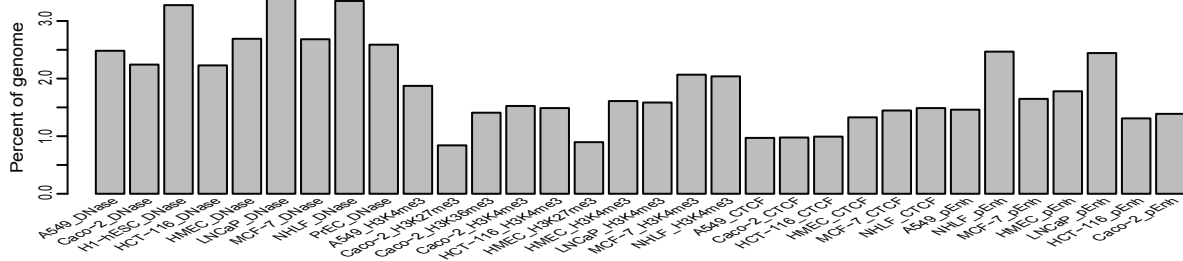

Supplement: Additional file 3 — An overview of the data depicting the percentages of overlapping regions between regulatory sequences among cell lines. The heatmap in part A shows the pair-wise percentages of overlapping regions for every feature pair. Features from associated cell types in our study are included. The strongest overlap is indicated in red and the weakest in blue, as depicted in the colour key. Features are clustered according to similarity in overlaps, and are labeled with the cell line names followed by the feature names. The “pEnh” term refers to putative enhancers. The percentage of the human genome covered by each feature is shown in part B. [file 1755-8794-7-34-S3.pdf]

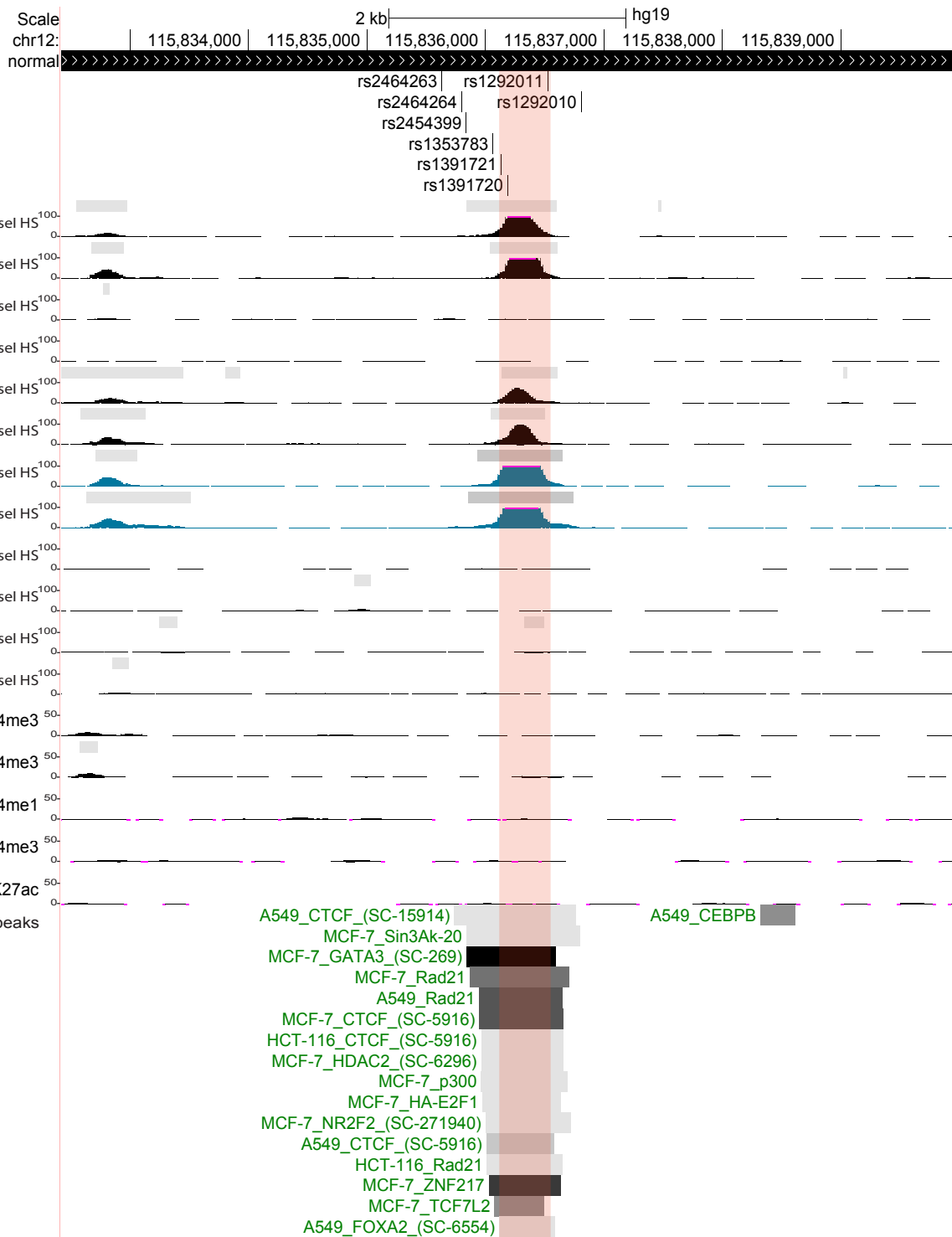

Supplement: Additional file 9 — Annotation features proximal to the rs1391720 SNP location from Breast.cancer LD80 set. The figure depicts annotation related to genetics, epigenetics, and TAF ChIP-seq peaks in proximity to the rs1391720 SNP in MCF-7 breast cancer and HMEC breast normal cell lines using the UCSC Genome Browser. The red vertical bar highlights the location of the 3 SNPs. From the top of the figure, the genetic information includes the locations of the SNPs and copy number status in MCF-7 cells, the SNPs are located in a gene desert. The chromatin information shows the DNase I hypersensitive sites in multiple cell types, occupancy sites of promoter marks in MCF-7 cells and active histone modification marks (H3K4me1, H3K4me3, H3K27ac) in HMEC cells. The ChIP-seq section shows the TAF-associated regions in cells we examined (where data is available). Hotspot of chromatin information and peaks in ChIP-seq section were reported by the ENCODE project with the gray scale color reflecting the magnitude of open chromatin and binding. [file 1755-8794-7-34-S9.pdf]

# Open Chromatin DNase-seq

# RNA-seq

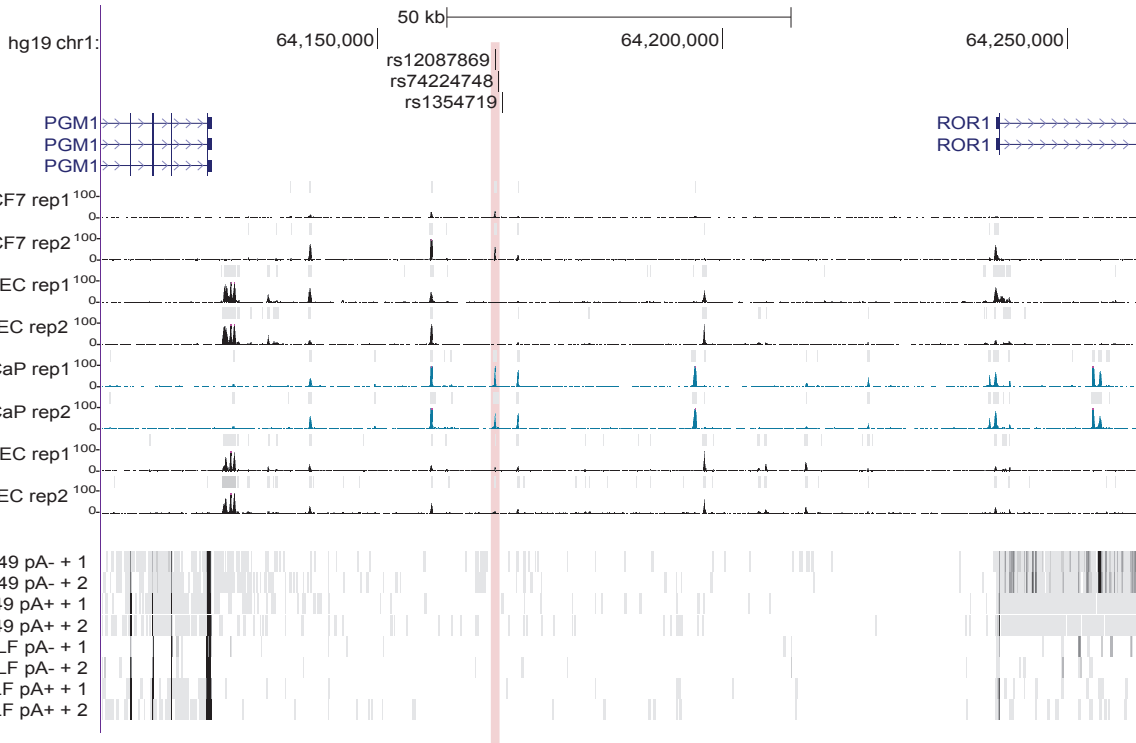

Supplement: Additional file 12 — Open chromatin features in other cell lines and expression data around rs12087869. The figure shows the open chromatin (DNase-seq) features in MCF7, HMEC, LNCaP and PrEC cell lines around the rs12087869 SNP. The RNA-seq signals on the plus DNA strand in A549 and NHLF cells are displayed. Both ROR1 and PGM1 genes are expressed in both cell types, and are not differentially expressed comparing between cancer and normal cells (as reported in Additional file 6). [file 1755-8794-7-34-S12.pdf]
