# Supplementary material for: On the identification of potential regulatory variants within genome wide association candidate SNP sets
Source: BMC Med Genomics. 2014 Jun 11;7:34. doi: 10.1186/1755-8794-7-34 (PMC4066296; doi:10.1186/1755-8794-7-34)
Supplement: Additional file 5 — Heatmap illustration of enrichment of LD95 SNPs in regulatory sequences. The figure displays the degrees of enrichment significance in regulatory sequences for GWAS SNPs extended to SNPs with r2 > =0.95 (LD95). The x-axis represents the LD95 SNP sets, and the y-axis represents data features from all cell types examined. Vertical and horizontal side bars are colored according to tissue types and whether it is data from a cancer or normal cell line. The enrichment of SNP lists within each feature is colored with a transformed value from multiple hypothesis adjusted q-values: -1x(log10 (q-values +0.0001)). Highly enriched feature and SNP list pairs are colored in yellow, and non-enriched pairs are colored in red. [file 1755-8794-7-34-S5.pdf]

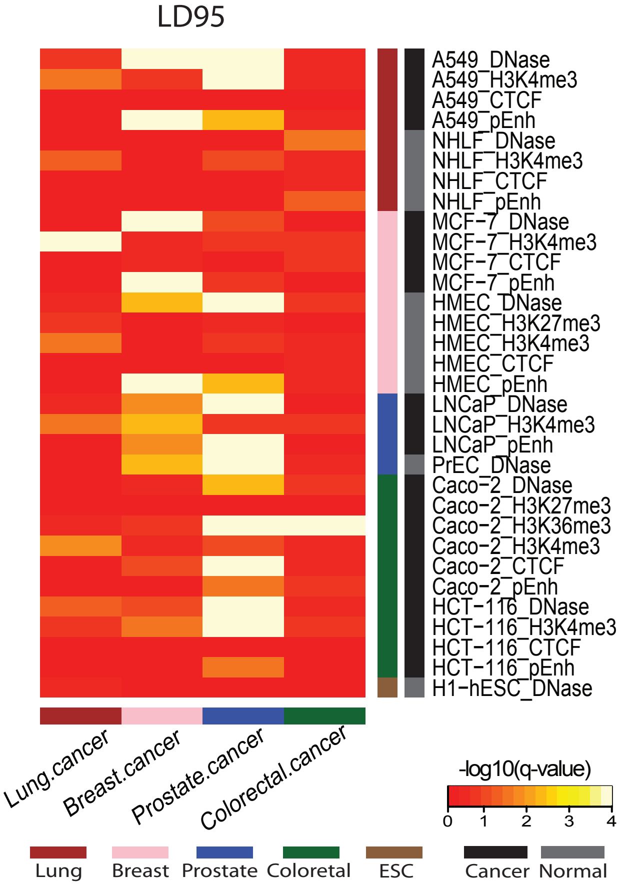
LD95
A549_DNase
A549_H3K4me3
A549_CTCF
A549_pEnh
NHLF_DNase
NHLF_H3K4me3
NHLF_CTCF
NHLF_pEnh
MCF-7_DNase
MCF-7_H3K4me3
MCF-7_CTCF
MCF-7_pEnh
HMEC_DNase
HMEC_H3K27me3
HMEC_H3K4me3
HMEC_CTCF
HMEC_pEnh
LNCaP_DNase
LNCaP_H3K4me3
LNCaP_pEnh
PrEC_DNase
Caco-2_DNase
Caco-2_H3K27me3
Caco-2_H3K36me3
Caco-2_H3K4me3
Caco-2_CTCF
Caco-2_pEnh
HCT-116_DNase
HCT-116_H3K4me3
HCT-116_CTCF
HCT-116_pEnh
H1-hESC_DNase
Lung.cancer
Breast.cancer
Prostate.cancer
Colorectal.cancer
-log10(q-value)
0
1
2
3
4
Lung
Breast
Prostate
Coloretal
ESC
Cancer
Normal
