# Supplementary material for: On the identification of potential regulatory variants within genome wide association candidate SNP sets
Source: BMC Med Genomics. 2014 Jun 11;7:34. doi: 10.1186/1755-8794-7-34 (PMC4066296; doi:10.1186/1755-8794-7-34)
Supplement: Additional file 7 — SNP prioritizing plots of Prostate.cancer and Colorectal.cancer LD80 SNPs. The file includes plots on differences in regulatory potential and allelic TF binding affinity (A-D) as well as TAF ChIP-seq data (E-F) for Prostate.cancer and Colorectal.cancer LD80 SNPs in addition to the Lung.cancer and Breast.cancer LD80 SNP sets plotted in Figures 4 and 5. [file 1755-8794-7-34-S7.pdf]

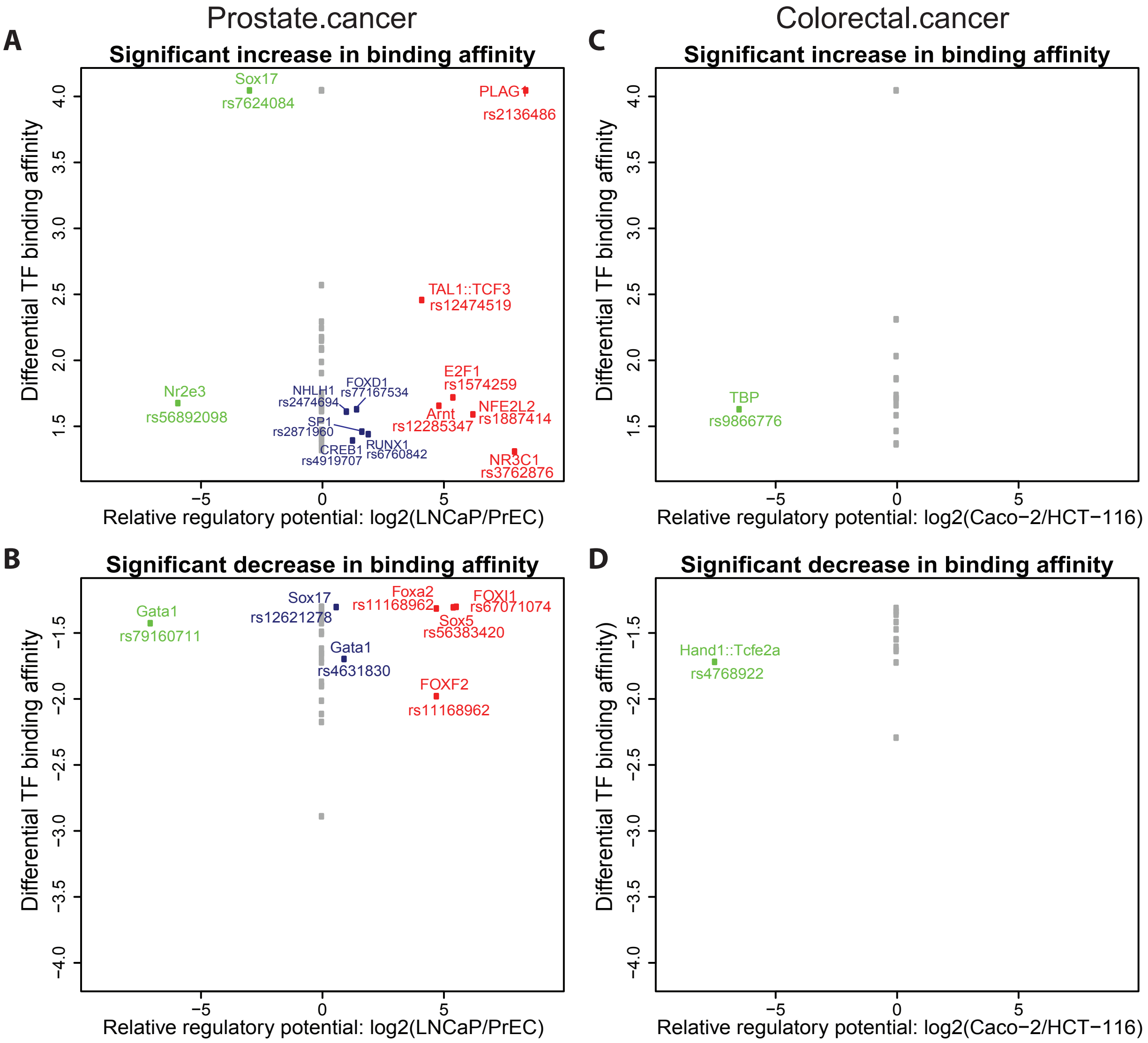

Prostate.cancer
Colorectal.cancer
A
Significant increase in binding affinity
Differential TF binding affinity
Relative regulatory potential: log2(LNCaP/PrEC)
Sox17
rs7624084
PLAG1
rs2136486
TAL1::TCF3
rs12474519
E2F1
rs1574259
Nr2e3
rs56892098
FOXD1
rs77167534
NHLH1
rs2474694
Arnt
rs12285347
NFE2L2
rs1887414
SP1
rs2871960
RUNX1
rs6760842
CREB1
rs4919707
NR3C1
rs3762876
C
Significant increase in binding affinity
Differential TF binding affinity
Relative regulatory potential: log2(Caco−2/HCT−116)
TBP
rs9866776
B
Significant decrease in binding affinity
Differential TF binding affinity
Relative regulatory potential: log2(LNCaP/PrEC)
Gata1
rs79160711
Sox17
rs12621278
Foxa2
rs11168962
FOXI1
rs67071074
Sox5
rs56383420
Gata1
rs4631830
FOXF2
rs11168962
D
Significant decrease in binding affinity
Differential TF binding affinity)
Relative regulatory potential: log2(Caco−2/HCT−116)
Hand1::Tcfe2a
rs4768922

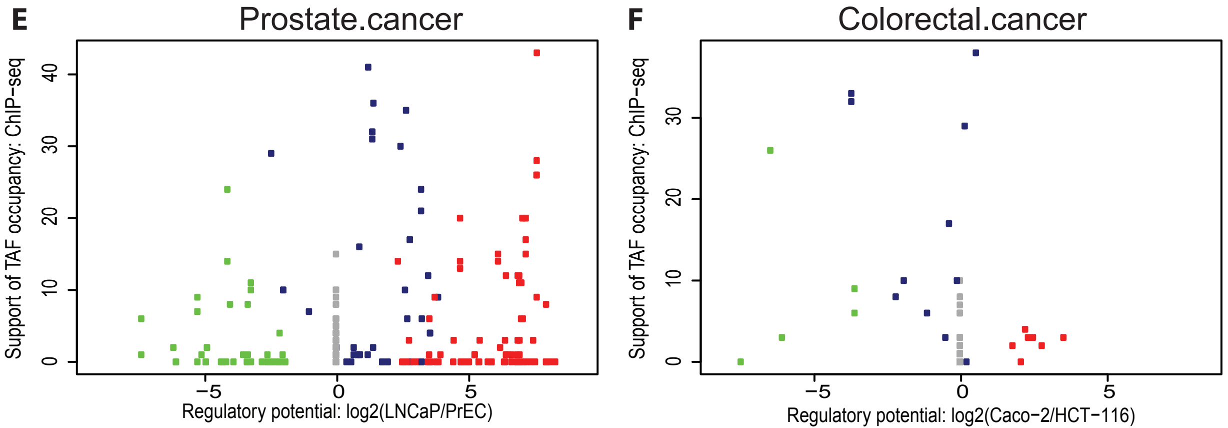
E
Prostate.cancer
Support of TAF occupancy: ChIP−seq
0
10
20
30
40
−5
0
5
Regulatory potential: log2(LNCaP/PrEC)
F
Colorectal.cancer
Support of TAF occupancy: ChIP−seq
0
10
20
30
−5
0
5
Regulatory potential: log2(Caco−2/HCT−116)
