# Supplementary material for: On the identification of potential regulatory variants within genome wide association candidate SNP sets
Source: BMC Med Genomics. 2014 Jun 11;7:34. doi: 10.1186/1755-8794-7-34 (PMC4066296; doi:10.1186/1755-8794-7-34)
Supplement: Additional file 10 — SNP prioritizing plots of Lung.Meta LD80 SNPs from the case study. The file includes plots displaying differences in regulatory potential and allelic TF binding affinity (A&B) as well as TAF ChIP-seq data (C) for Lung.Meta LD80 SNPs corresponding to Figures 4 and 5. [file 1755-8794-7-34-S10.pdf]

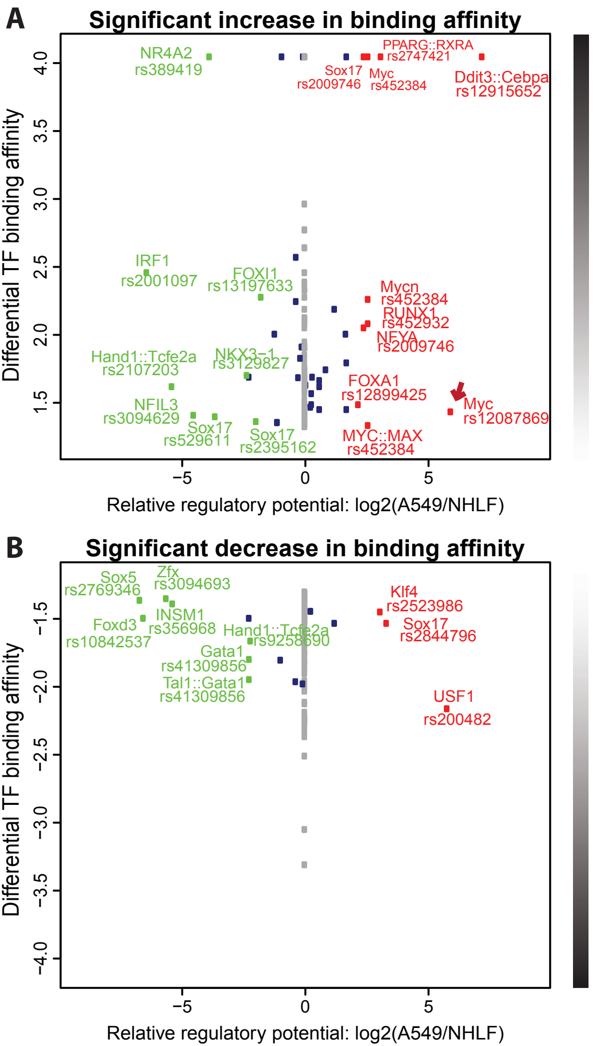
A
Significant increase in binding affinity
NR4A2
rs389419
PPARG::RXRA
rs2747421
Sox17
rs2009746
Myc
rs452384
Ddit3::Cebpa
rs12915652
IRF1
rs2001097
FOXI1
rs13197633
Mycn
rs452384
RUNX1
rs452932
NFYA
rs2009746
Hand1::Tcfe2a
rs2107203
NKX3−1
rs3129827
FOXA1
rs12899425
Myc
rs12087869
NFIL3
rs3094629
Sox17
rs529611
Sox17
rs2395162
MYC::MAX
rs452384
4.0
3.5
3.0
2.5
2.0
1.5
−5
0
5
Differential TF binding affinity
Relative regulatory potential: log2(A549/NHLF)
B
Significant decrease in binding affinity
Sox5
rs2769346
Zfx
rs3094693
INSM1
rs356968
Foxd3
rs10842537
Hand1::Tcfe2a
rs9258690
Gata1
rs41309856
Tal1::Gata1
rs41309856
Klf4
rs2523986
Sox17
rs2844796
USF1
rs200482
−1.5
−2.0
−2.5
−3.0
−3.5
−4.0
−5
0
5
Differential TF binding affinity
Relative regulatory potential: log2(A549/NHLF)

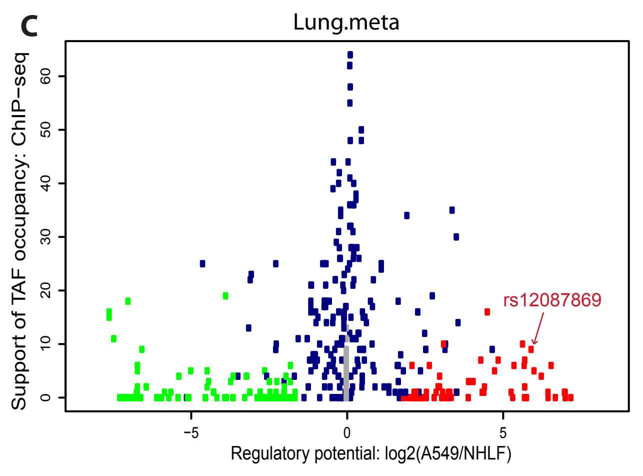

C
Lung.meta
Support of TAF occupancy: ChIP−seq
0
10
20
30
40
50
60
−5
0
5
Regulatory potential: log2(A549/NHLF)
rs12087869
