# Supplementary material for: On the identification of potential regulatory variants within genome wide association candidate SNP sets
Source: BMC Med Genomics. 2014 Jun 11;7:34. doi: 10.1186/1755-8794-7-34 (PMC4066296; doi:10.1186/1755-8794-7-34)
Supplement: Additional file 14 — Two-dimensional heatmap of chromatin interaction in the neighbourhood of the rs1391720 SNP. The figure shows Hi-C chromatin interaction datasets in H1 human ES cells (upper) and IMR90 fibroblast cells (lower panel) obtained from Dixon et al. [31] in the neighbourhood of the rs1391720 SNP and two other SNPs in high LD. The SNPs overlapping the potential cancer-specific enhancer and over 16 TAF ChIP-seq data are labeled in the middle panel along with genes from UCSC and transcripts from Ensembl that include long non-coding RNAs. The topological domains (TADs) from both cell types were shown to indicate genomic neighbourhood of stronger within-domain interactions. The heatmap values indicated in a color scale correspond to the number of times that reads in two 20 kb bins were sequenced as a pair, with the red color indicating stronger interaction and white being little or no interaction. The 85 percentile read counts (22 for H1 and 18 for IMR90 cells) were used as the upper limit for the heatmap to avoid color domination of extremely interactive regions. This plot was generated using ‘HiTC’ R package, and the dotted lines were drawn to aid in visualizing the interactive domain in which the SNP is located. The TAD region (from H1 cells) containing the SNP is highlighted in a light pink box. [file 1755-8794-7-34-S14.pdf]

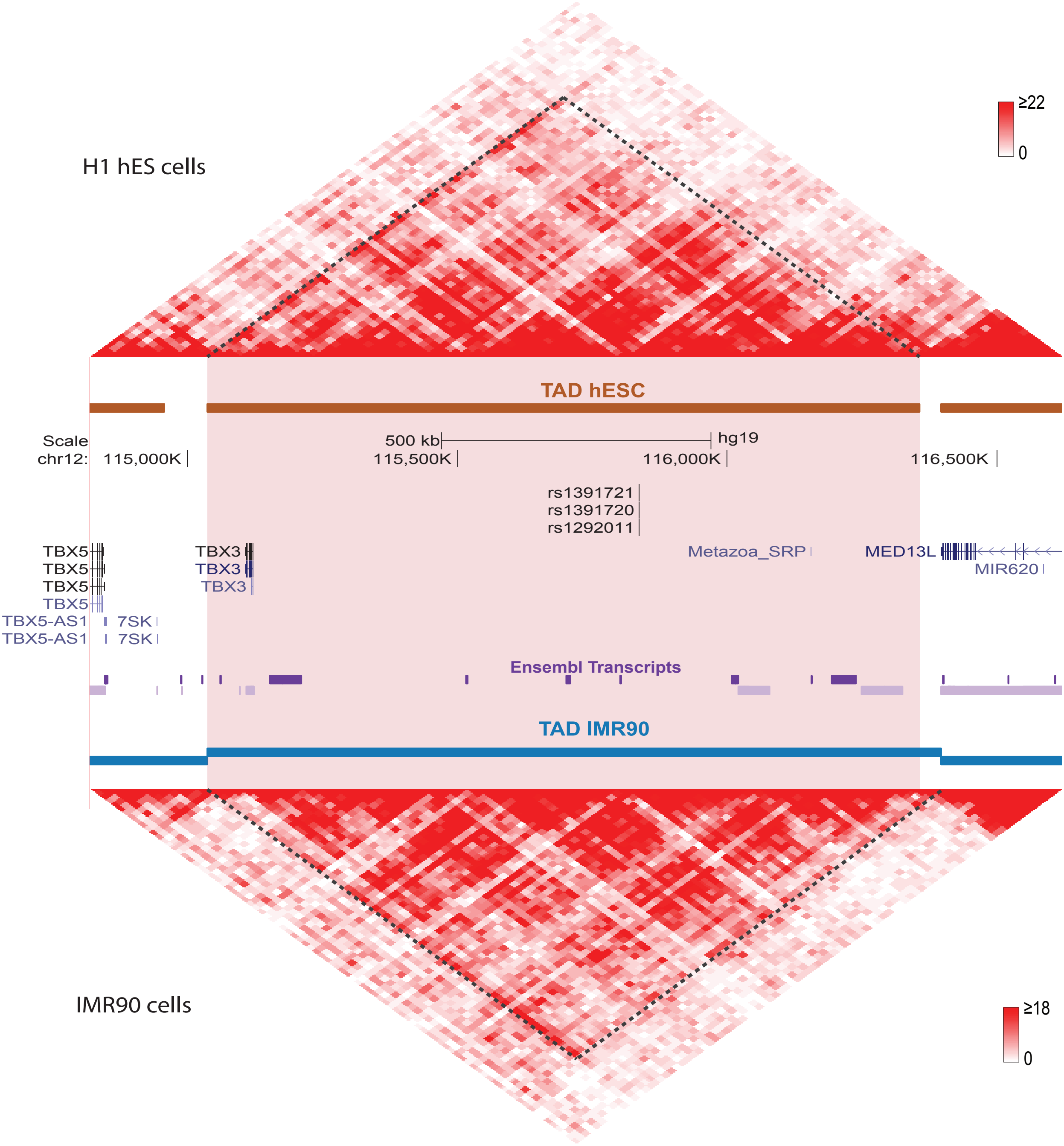

H1 hES cells
≥22
0
TAD hESC
Scale
500 kb
hg19
chr12:
115,000K
115,500K
116,000K
116,500K
rs1391721
rs1391720
rs1292011
TBX5
TBX5
TBX5
TBX5
TBX5-AS1
TBX5-AS1
7SK
7SK
TBX3
TBX3
TBX3
Metazoa_SRP
MED13L
MIR620
Ensembl Transcripts
TAD IMR90
IMR90 cells
≥18
0
