# Supplementary material for: On the identification of potential regulatory variants within genome wide association candidate SNP sets
Source: BMC Med Genomics. 2014 Jun 11;7:34. doi: 10.1186/1755-8794-7-34 (PMC4066296; doi:10.1186/1755-8794-7-34)
Supplement: Additional file 4 — Enrichment of GWAS SNPs in all HTS data surveyed. This illustration provides an alternative presentation of the enrichment result shown in Figure 3. Figures exhibit the overlap counts of LD80 SNPs obtained from Lung (A), Breast (B), Prostate (C) and Colorectal (D) cancer GWAS with regulatory sequences in all cell types examined. The x-axis indicates the cells of origin and feature data sets that reflects regulatory sequences (all from the ENCODE consortium). The y-axis shows the number of SNPs in the set that are within each category of regulatory sequences. The “pEnh” term refers to putative enhancers. The red dots represent the actual overlapping counts of the SNP sets with regulatory sequences, and the boxplots represent the distributions of overlapping counts by randomly selected SNP sets with matching minor allele frequencies, GC content (+/-500 bps) and distance to the nearest TSSs repeated 1000 times. Multiple hypothesis-adjusted q-values lower than 0.05 are noted above the boxplots. [file 1755-8794-7-34-S4.pdf]

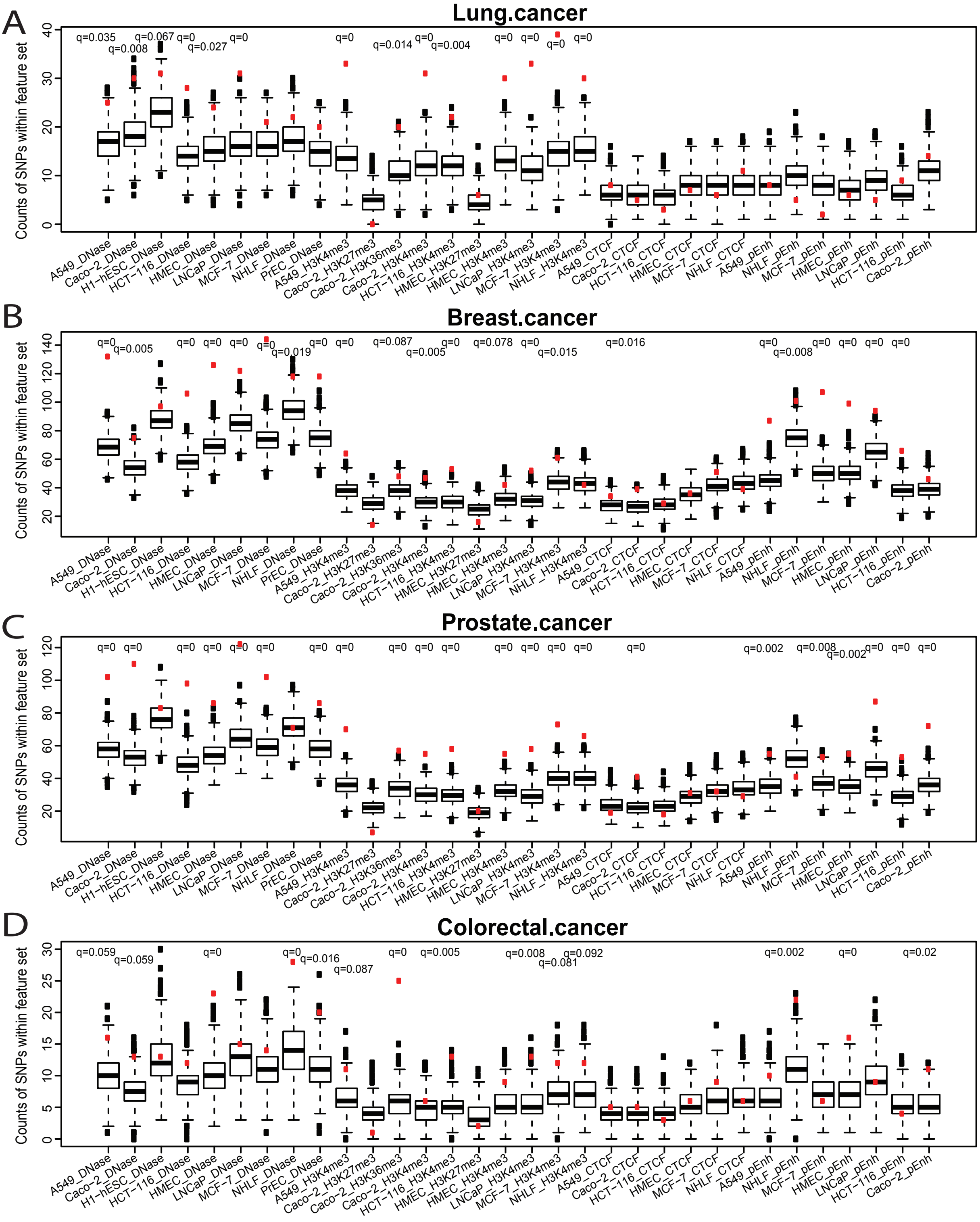

A
Lung.cancer
Counts of SNPs within feature set
0
10
20
30
40
q=0.035 q=0.008 q=0.067 q=0 q=0.027 q=0 q=0 q=0.014 q=0 q=0.004 q=0 q=0 q=0 q=0
A549_DNase
Caco-2_DNase
H1-hESC_DNase
HCT-116_DNase
HMEC_DNase
LNCaP_DNase
MCF-7_DNase
NHLF_DNase
PrEC_DNase
A549_H3K4me3
Caco-2_H3K27me3
Caco-2_H3K36me3
Caco-2_H3K4me3
HCT-116_H3K4me3
HMEC_H3K27me3
HMEC_H3K4me3
LNCaP_H3K4me3
MCF-7_H3K4me3
NHLF_H3K4me3
A549_CTCF
Caco-2_CTCF
HCT-116_CTCF
HMEC_CTCF
MCF-7_CTCF
NHLF_CTCF
A549_pEnh
NHLF_pEnh
MCF-7_pEnh
HMEC_pEnh
LNCaP_pEnh
HCT-116_pEnh
Caco-2_pEnh
B
Breast.cancer
Counts of SNPs within feature set
20
40
60
80
100
120
140
q=0 q=0.005 q=0 q=0 q=0 q=0 q=0.019 q=0 q=0 q=0.087 q=0.005 q=0 q=0.078 q=0 q=0.015 q=0.016 q=0 q=0.008 q=0 q=0 q=0 q=0
C
Prostate.cancer
Counts of SNPs within feature set
20
40
60
80
100
120
q=0 q=0 q=0 q=0 q=0 q=0 q=0 q=0 q=0 q=0 q=0 q=0 q=0 q=0 q=0 q=0 q=0.002 q=0.008 q=0.002 q=0 q=0 q=0
D
Colorectal.cancer
Counts of SNPs within feature set
0
5
10
15
20
25
30
q=0.059 q=0.059 q=0 q=0 q=0.016 q=0.087 q=0 q=0.005 q=0.008 q=0.081 q=0.092 q=0.002 q=0 q=0.02
